# Linking signaling dynamics and cell fate decisions through single-cell imaging: evidence and challenges

**DOI:** 10.3389/fcell.2025.1656051

**Published:** 2025-10-23

**Authors:** Fulvio Bonsignore, Sara Pozzi, Erika Aloi, Davide Mazza, Samuel Zambrano

**Affiliations:** ^1^ Experimental Imaging Center, IRCCS San Raffaele Scientific Institute, Milan, Italy; ^2^ School of Medicine, Vita-Salute San Raffaele University, Milan, Italy; ^3^ Division of Genetics and Cell Biology, IRCCS San Raffaele Scientific Institute, Milan, Italy

**Keywords:** signaling dynamics, cell fate decisions, live-cell microscopy, NF-kB, p53, MAPK, Hes1

## Abstract

Our ever-growing capacity to observe dynamic processes at the single-cell level has highlighted how cells use complex signaling dynamics to provide adequate responses to intra- and extracellular cues. Specifically, there is increasing evidence that signaling dynamics can be functional in determining cell fate decisions. In this work, we provide an overview of the growing body of evidence supporting this idea across diverse biological contexts—including immune responses, reactions to DNA damage and growth factors, and embryonic development. In doing so, we aim to provide a precise conceptualization of what is meant when we say that signaling dynamics can determine cell fate, a unifying view of the methodologies used to sustain this claim and to identify some of the existing gaps in our mechanistic understanding of this process. We believe that the body of work hereby described strongly supports the importance of considering the temporal dimension of signaling when seeking to understand how cellular responses are regulated.

## 1 Introduction

Single-cell technologies, specifically microscopy and live cell imaging (Shroff et al., 2024), have provided us unprecedented insights on the fascinating dynamics of biological processes. In this context, some of the most surprising observations have emerged by the analysis of signaling dynamics, i.e., the temporal evolution of the processes by which cells provide responses to stimuli. A naif point of view suggests that signaling systems would simply become “active” as long as the cues persist in a stimulus-specific way. This kind of specificity can indeed be attained by a sufficiently large combination of receptors and ligands (Su et al., 2022). Instead, imaging of the activity of key proteins involved in the signaling pathways like kinases (Regot et al., 2014) or transcription factors (TFs) (Liu and Tjian, 2018) has shown that signaling systems do not simply switch from an inactive state to an active one, but rather they display a surprising variety of dynamic behaviours in response to different stimuli, involved in processes like the immune response (Nelson et al., 2004), DNA damage response (Geva-Zatorsky et al., 2006) and development (Shimojo et al., 2008), to cite a few. This observation raised the possibility that the complex signaling dynamics observed in an increasing number of systems (Levine et al., 2013) could be an efficient mechanism to minimize errors and improve information transmission in the inherently stochastic cellular environment (Selimkhanov et al., 2014). Furthermore, these dynamics can provide novel ways to understand how cells can decode information through different downstream mechanisms (Purvis and Lahav, 2013), considering that signaling pathways often display a bow-tie structure (Itoh et al., 2024), where the information from multiple signals converge on few key transducers to produce specific outcomes.

Although the precise molecular mechanisms by which signaling dynamics might exert their function is not yet fully elucidated (Meyer et al., 2025), in the past decade the integration of imaging data with transcriptomics, genetic perturbations and functional assays have converged to a common view by which signaling dynamics can “determine cell fate decisions” (Simon et al., 2024). The goal of this review is first to try to put together in a coherent corpus the wide body of work supporting this claim that has been published in the past years in selected biological processes. Before doing so, though, it is reasonable to delineate a working definition of “determinism” when speaking about cells, and of what is meant by “cell fate” in a way that encompasses the relevant literature in this context.

In classical physics terms, determinism is a consequence of the laws of dynamics: the evolution of an isolated system can be completely predicted, given a precise knowledge of the position and the velocities of every single atom composing the system and their interactions. In cell biology, a more loose definition is sufficient: even for a couple of cells that we could consider “identical”, we would expect a certain degree of variability in the number, position and motion of the macromolecules that compose them - because of the inherent uncertainty of their underlying physics (e.g., thermal effects that make single molecule motion unpredictable in practical terms) and the high degree of complexity of these systems. And yet, we would expect that these cells should behave predictably and consistently to a certain extent, since in different biological processes they need to provide context-dependent responses to specific inputs. In other words, we expect these systems to have some intrinsic buffering capabilities that allow multiple similar (but microscopically distinct) initial states to converge, in a “deterministic” way, to a certain output state (Symmons and Raj, 2016). A clear example comes from embryonic development, where a zygote generates a whole organism with roughly a billion different cells that specialize to specific subtypes [more than 200 in humans (Milo and Phillips, 2016)]. This precise choreography is the result of an evolutionary process that has selected for a precise set of regulatory mechanisms (Alon, 2007) able to produce a functional organism with widely different cell types (from neurons to gut cells). An analogous degree of predictability can also be expected in other situations, for example, where cells have to respond to a pathogen in an adequate way to avoid its proliferation. Hence, we can conclude that there is a scale of the description of a cell state upon which we might expect cells to be “deterministic” and hence predictable to some extent.

**FIGURE 1:**
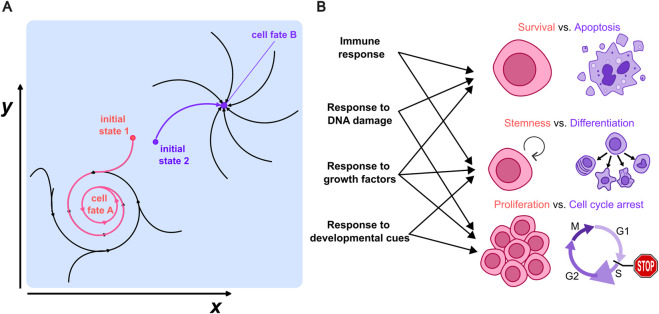
**(A)** Example of two possible attractors in the space of cell states (*x, y*) towards which a given cell can converge. Each corresponds to the fate of a cell upon a certain cue. **(B)** Examples of possible fates that go beyond the classic differentiation of cells taking place in development, to include the cell’s life-death or proliferation/cell cycle arrest decisions. Each of these cell fates could be mapped to a distinct attractor towards which the cell state can converge and might arise in different biological processes.

Development is also a useful starting point to provide a definition of cell fate: stem cells differentiate into hundreds of cell types, and each of these types correspond to a different fate. Mathematically, these cell fates can be understood as attractors (Casey et al., 2020), i.e., specific sets of cell states that define dynamic trajectories within the space of possible states towards which the state of a given cell can converge (Figure 1A); this dynamic point of view generalizes Waddington’s landscape notion (Waddington, 2016) to more dynamic “fates”, which could be characterized, e.g., by oscillating expression of certain markers (Figure 1A). A revision of the literature shows that the notion of cell fate goes beyond development to include also differentiation processes taking place on regenerating tissues (Massenet et al., 2021), hematopoiesis (Kao et al., 2024) and immune responses (Franz and Kagan, 2017); but this can be further generalized to changes in the cell state taking place in response to radiotherapy (Chen et al., 2022) or even in the response to different forms of stress (Hetz, 2012). Cell fate can hence be generalized from a theoretical point of view as any attractor towards which a set of cell states can converge such as survival, apoptosis, stemness or differentiation, proliferation or cell cycle arrest (Figure 1B). Since attractors are defined as states able to “attract” trajectories starting close to them, they are thought to be somehow robust to any perturbation that might change the system state slightly. From an experimental point of view, the fate of a cell, once determined, robustly resists small perturbations or fluctuations: cells will change their fate in a predictable way only if there is a sufficiently strong perturbation.

Considering these definitions, here we will review the literature investigating the connections between signalling dynamics and cell fate determination. We will highlight how heterogeneous responses can be leveraged to provide novel insights into this relation, thanks to the use of single-cell live imaging. We will focus on four biological contexts for which, in our view, this connection has been explored in greater detail: immune signaling, response to genotoxic stress, response to growth factors and development; in each of them we mainly focus on specific pathways that have been better characterized in the literature. We also aim to describe the challenges that need to be addressed to dissect the molecular mechanisms linking signaling dynamics to the determination of cell fate and discuss potential future methodological developments that might help in this quest.

## 2 From dynamics to cell fate in different biological contexts

### 2.1 Immune signaling: NF-κB and other related pathways

The innate and adaptive immune responses to threats in multicellular organisms include different forms of “cell fate decisions”. In this context, the NF-κB (Nuclear Factor Kappa-light-chain-enhancer of activated B cells) system is a key transcription factor family involved in regulating immune and inflammatory responses, cell survival, but also differentiation (Hayden and Ghosh, 2008). It is composed of five monomers: RelA (p65), RelB, c-Rel, p50, and p52, which form homo- or heterodimers with distinct functions. RelA, often paired with p50, plays a central role in the canonical NF-κB pathway, mediating inflammatory and immune responses through its strong TAD (Transactivation Domain); this is by far the subunit whose dynamics have been more thoroughly characterized (Kizilirmak et al., 2022) although recently growing attention is being paid to the dynamics of c-Rel (Martin et al., 2020), another potent transcription activator crucial in immune cell activation and proliferation.

**FIGURE 2:**
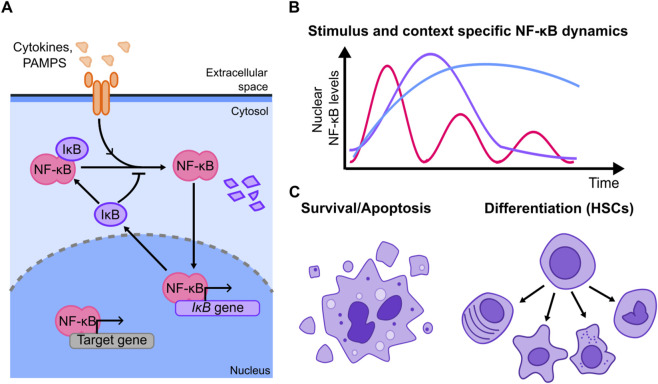
**(A)** Simplified scheme of the NF-κB signaling system showing its key negative feedback (other positive and negative feedbacks have been reported and are not shown). Stimulus triggers signaling cascades leading to the degradation of the inhibitors IκB that keep NF-κB sequestered in the cytosol, allowing NF-κB to localize into the nucleus. There, it drives the expression of its targets, which include the inhibitors themselves, which constitute a series of negative feedbacks. **(B)** Examples of the dynamics observed in the NF-κB signaling system; these dynamics are known to be stimulus and cell dependent to a large extent. **(C)** Cell fates that have been shown to be followed in an NF-κB dynamic-specific way, including cell death and differentiation into different hematopoietic cell types.

Population-level experiments (sometimes coupled with mathematical models) allowed the dissection of different mechanisms of regulation of NF-κB upon stimuli, including different pathways of activation and mechanisms of regulation (Hoffmann et al., 2002; Covert et al., 2005; Werner et al., 2005; 2008). These studies showed that the canonical NF-κB signaling is finely controlled by a system of specific activators and negative feedbacks (Figure 2A): dimers including RelA are kept inactive by inhibitors of the IκB family, which sequester them in the cytoplasm under resting conditions. Activation through stimuli such as cytokines or microbial products leads to IκB phosphorylation and degradation, allowing NF-κB dimers to translocate to the nucleus (Hoffmann et al., 2002), where they also drive the expression of IκB and other negative regulators like A20 (Werner et al., 2008; Ashall et al., 2009). Given the central role played by the NF-κB in innate immune responses, this was one of the first signalling systems to be studied through single cell-live cell imaging of a fluorescently tagged version of RelA (Nelson et al., 2004). This study revealed that this system can give rise to a wealth of nuclear localization dynamics of RelA, heterogeneous even across homogeneous cell populations, and including oscillations with a period close to 1.5 h (Nelson et al., 2004) (Figure 2B). Since then, different studies have used live-cell imaging to gain insights in NF-κB function, as reviewed in (Kizilirmak et al., 2022): NF-κB dynamics have been shown to control gene expression (Nelson et al., 2004; Ashall et al., 2009; Tay et al., 2010) highlighting that genes belonging to different functional classes respond to NF-κB oscillations by accumulating at different rates (Zambrano et al., 2016). Subsequently, several efforts have been devoted to understand if this behavior can indeed have specific functional consequences and drive different cell fate decisions.

Since NF-κB is a central regulator of anti-apoptotic pathways [and hence is dysregulated in cancer (Ben-Neriah and Karin, 2011)] some studies have tried to connect its dynamics with life-death decisions in cells (Figure 2C). For example, using a microfluidic device designed to deliver short pulses of TNF-ɑ, Lee and colleagues demonstrated that there was a duration of the TNF-ɑ stimulation by which the response of NF-κB optimally counter-balanced the pro-apoptotic pathways elicited by this same cytokine (Lee et al., 2016). However, the precise molecular mechanism remained to be dissected and the correlation between dynamics and cell fate was not explored at the single cell level. Evidence pointing to the same direction arose studying the necroptosis mechanism in mouse L929 fibroblasts (Metzig et al., 2020). There, it was found that the rate of death upon TNF-ɑ could not be due to a direct activation of apoptotic pathways, but instead shall be modulated by the concomitant activation of NF-κB forming an incoherent feed forward loop. Although - also in this case - a direct correlation between dynamics and cell fate is not established at the single cell level, perturbation of the NF-κB activation mechanism through specific silencing of negative regulators changed the fraction of cells undergoing apoptosis, providing evidence that cell death is indeed affected by NF-κB dynamics.

Other works have shown that NF-κB can play a role in cell differentiation, for example, in hematopoietic stem cells (Figure 2C). Using a knock-in mouse model (Kull et al., 2022) highlighted that NF-κB dynamics change throughout the hematopoietic hierarchy. Combining imaging with advanced microfluidics they show at the single cell level that cells with different types of NF-κB dynamics are more likely to differentiate to certain cell types. Furthermore, the expression of some lineage-specific genes depends on NF-κB dynamics, providing plausible mechanisms linking signaling dynamics to cell fate. This observation was further validated by showing that “forcing” NF-κB to oscillate with pulses of TNF-ɑ can skew cell fate decisions promoting differentiation into specific cell types. This functional connection between dynamics and differentiation is in line with a recent study (Singh et al., 2025) showing that HSC differentiation is skewed in mouse models with specific knock-outs of the inhibitor proteins IκBs, which in turn produces different NF-κB dynamic responses that correlate with higher inflammation levels (although a specific single-cell connection between NF-κB dynamics and differentiation is not experimentally established). Interestingly, a separate *in vivo* study highlights that the establishment of the hematopoietic compartment in zebrafish (Campbell et al., 2024) requires two pulses of RelA activity, which are separated by 12 h (much slower than what is observed upon acute inflammatory signals). NF-κB regulation of the cell cycle is fundamental here, and drug-mediated inhibition of NF-κB provides additional confirmation of these results.

It is not surprising that NF-κB has also been proposed to play a role in other hematopoiesis-derived cells. In B lymphocytes bulk measurements of NF-κB dynamics suggest that oscillations might be important for the specific expression of CD83, a marker of B cell maturation (Inoue et al., 2016). More recently, a study (Mitchell et al., 2018) shows that B cell proliferation presumably relies on an NF-κB circuit that regulates dynamically apoptosis and cell cycle genes; further, the dynamics of c-Rel govern the transition of B cells from a proliferative state to plasma cell differentiation (Roy et al., 2019). In these two studies, though, a precise characterization of how NF-κB dynamics control of the cell cycle at single cell level has not been carried out, although evidence of their mutual regulation has been provided through live cell imaging (Ankers et al., 2016) and would deserve further characterization.

NF-κB dynamics have also been suggested to play a role in the fate decisions of myeloid cells. Machine learning approaches combined with live cell imaging show that macrophages can distinguish ligands through signaling dynamics (Adelaja et al., 2021) in a polarization-dependent way (Singh et al., 2024). It is hence tempting to speculate that this would determine subsequent functional effects in macrophages upon stimuli. Importantly, response specificity might be enhanced by c-Rel and Rel-A dynamics simultaneously elicited by different stimuli, as shown in doubly fluorescently tagged macrophages (Rahman et al., 2024). Evidence from cell lines obtained by the same group indeed suggests that specific gene expression programs are elicited by RelA and c-Rel dynamics (Martin et al., 2020), but the specific dynamic-dependent transcriptional control mechanism remains yet to be fully characterized. We cannot exclude though that the dynamics of NF-κB play side roles beyond transcription of direct targets, for example on chromatin, where NF-κB dynamics can elicit long-term epigenomic reprogramming (Cheng et al., 2021) as a reinforcing mechanism to induce specific gene expression programs.

Finally, we have to point out that other TFs can be concomitantly activated with NF-κB in innate and adaptive immune responses, which could contribute to further refine the cell fate decision-making process. An example is NFAT (Nuclear Factor of Activated T-cells), whose subunits display different dynamics (Yissachar et al., 2013) and has been shown to display a combinatorial activation alongside NF-κB at single cell level upon different stimuli, leading to specific gene expression programs (Huang et al., 2023). Other important immune- and differentiation-related signal transducers are JAK-STAT, for which studies have shown that the phosphorylation dynamics determine cell fate decisions in erythropoiesis (Adlung et al., 2021). Here, however, the specific single-cell dynamics have not been yet described nor the effect of the simultaneous activation of NF-κB, which might provide further insights considering its role in blood cell differentiation, as delineated above.

Overall the dynamics of NF-κB are probably some of the most well-characterized in mammalian signaling systems, where strong evidence points for a role of its dynamics governing cell fate decisions. This seems to largely rely on NF-κB dynamics-specific control of gene expression signatures. However, the dynamics-cell fate relation has not always been studied at single cell level and remains to be fully dissected.

### 2.2 Response to genotoxic stress: p53

**FIGURE 3:**
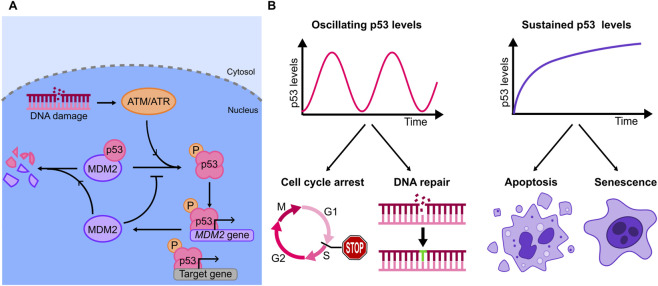
**(A)** Diagram of the principal elements of the p53 genetic circuit, involving the kinases ATM/ATR, and the key negative feedback, MDM2, that contributes to p53 degradation. **(B)** Examples of oscillatory and sustained dynamics observed for p53 upon specific stimuli (top), and corresponding outcomes in terms of cell fates (bottom): increasing evidence shows that oscillations are related with cell cycle arrest and DNA repair, while sustained dynamics can lead to apoptosis and senescence.

p53, also known as “the guardian of the genome”, has a central role in the response to genotoxic stress signals (Kuerbitz et al., 1992). p53 expression is tightly regulated, as demonstrated by landmark early work that highlighted the central role of the protein MDM2 (Mouse Double Minute 2) in the control of p53 activity (Harris and Levine, 2005). In normal conditions, MDM2 targets p53 for degradation, keeping its levels low (Momand et al., 1992; Haupt et al., 1997) (Figure 3A). Following activation, DNA damage sensing kinases (e.g., ATM and ATR) phosphorylate the p53-MDM2 complex, leading to p53 accumulation in the nucleus. Active p53 tetramerizes and binds to promoters and enhancers of its target genes to activate their transcription (Stommel and Wahl, 2004) (Figure 3A). Importantly, the genes targeted by p53 have different (and sometimes opposite) roles in the response to genotoxic stress, with functions that range from cell cycle arrest and DNA repair to the promotion of terminal fates such as cell senescence and apoptosis. MDM2 is itself a transcriptional target of p53, resulting in a negative feedback loop that generates diverse dynamics of p53 expression levels: the richness of these dynamics - that include monotonic increase in p53 level, single analogue pulses or digital oscillations (Figure 3B), can only be appreciated by analyses at the single cell level, as bulk experiments - averaging together dynamics that are partially asynchronous - blur these sharp responses.

For example, upon ionizing radiation, fluorescently labeled p53 in MCF-7 cells displays oscillatory dynamics, with a period (∼5.5 h) and amplitude that are independent from the irradiation dose (Lahav et al., 2004). Progressive loss of synchronicity of the p53 pulses between cells results in apparent damped oscillations at the population level. Notably, in unstressed conditions, proliferating MCF7 cells display spontaneous isolated pulses of p53, due to replication-induced DNA damage, that are however incapable of eliciting a transcriptional response (Loewer et al., 2010). Sustained activation is therefore required to evoke the p53 transcriptional activity.

Further studies highlighted that p53 dynamics can widely vary in a context-specific manner: either oscillations, single pulses or sustained responses can be observed, depending on the stimulus and on the cellular model used (Batchelor et al., 2011; Stewart-Ornstein and Lahav, 2017). This variety of responses are due to the specific activation of additional p53 regulators, such as the phosphatase Wip1, that by dephosphorylating p53 enhances pulsatile dynamics.

The stimulus specificity in p53 dynamics suggested a biological function. The first direct evidence that different p53 dynamics can dictate distinct cell fates was provided by (Purvis et al., 2012). To this end the authors used a small-molecule inhibitor of the p53-MDM2 interaction, Nutlin-3a, to convert the oscillating p53 dynamics observed upon γ-irradiation into a sustained one: genes involved in cell cycle arrest were activated by both oscillating and sustained dynamics, while genes associated to senescence were more robustly upregulated by sustained p53 accumulation. Accordingly, sustained p53 dynamics resulted in a higher fraction of cells undergoing senescence, as proved by a senescence-associated *β*-galactosidase assay (Figure 3B).

Further work confirmed that p53 oscillations might promote pro-survival programs. Total-body irradiation of mice revealed that p53 dynamics vary across tissues: radioresistant tissues (intestine) display p53 oscillations, while radiosensitive ones (thymus, spleen) display sustained dynamics (Stewart-Ornstein et al., 2021). Similar observations have been made in cellular models of human cancers: cancer cell lines responding to the genotoxic chemotherapy Etoposide with p53 oscillations are less sensitive than those displaying a monotonic increase in p53 levels (Yang et al., 2018). Differences in upstream signaling, such as ATM-controlled MDM2 degradation, was found as the leading cause of the diverse p53 dynamics across these cell lines. How cells then decode different dynamics into diverse responses is instead less clearly understood.

As suggested by the original work of (Purvis et al., 2012) such decoding possibly occurs downstream, at the level of the p53 target gene expression. mRNAs with fast degradation rates might not be able to accumulate when p53 oscillates, but they could upon sustained p53 activity. Similarly, the degradation rate of protein products dictates their abundance depending on the upstream signaling dynamics. Along these lines, a work focusing on p21 (a major p53 target) showed that how fast p21 accumulates in early phases post-treatment skews cell fate to either proliferation or senescence (Hsu et al., 2019). More generally, however, while it is now clear that both mRNA (Porter et al., 2016) and protein stability of p53 targets widely vary across p53 target genes (Hanson et al., 2019), no direct association between the targets lifetime and their functional role (e.g., pro-survival or pro-apoptotic) has clearly emerged.

Decoding of dynamics into different fates might also be carried out by ‘kinetic competition’ between different pathways. An example is provided by the work by (Paek et al., 2016) which investigated the role of p53 dynamics in determining why only a fraction of cells undergo apoptosis following treatment with the genotoxic chemotherapy cisplatin. The authors found that only those cells accumulating p53 early and at a fast rate were those undergoing apoptosis. Concurrently to the increase of p53 levels, cisplatin causes also the accumulation of IAP (Inhibitors of Apoptosis) genes, that set a progressively increasing threshold that p53 needs to overcome to induce cell death: if p53 increases faster than IAP, cells go through apoptosis, if p53 accumulates slowly and does not overcome the threshold, the cell survives. These findings - that could not have been obtained with bulk assays which average together the dynamics of cells with divergent cell fates - were further supported by a theoretical paper in which the authors model how the interplay of p53 dynamics and the XIAP (X-linked Inhibitor of Apoptosis) induction rate turned out to be critical to determine the cancer cells therapeutic response (Abukwaik et al., 2023). As multiple pathways can be simultaneously activated by genotoxic stress, it is likely that more insights on determinants of cell fate could arise from studies that investigate the cross-talk between p53 dynamics and those of other signaling molecules, such as NF-κB (Konrath et al., 2020) and FOXO1 (Jose et al., 2024). Along these lines, these cross-talks might explain why timing of combinatorial treatments have an impact on the cell fate outcome (Chen et al. 2016).

Finally, it is possible that p53 dynamics could control cell fate through mechanisms that are unrelated to its transcriptional activity. A recent mathematical modelling work suggests that oscillations might allow p53 to redistribute across sites where it needs to exert some non-canonical functions (Heltberg et al., 2022), such as facilitating the recruitment of DNA-repair machinery at damaged hubs (Wang et al., 2022). Accordingly, p53 oscillations appear to result in more efficient DNA-repair than sustained dynamics when measured experimentally (Heltberg et al., 2022).

To summarize, the dynamics of activation of p53 have been found to correlate with the determination of cell fates in different models and through different techniques. The studies investigating this connection have identified multiple putative mechanisms, including gene-specific responses, competition with other pathways and non-canonical p53 activities. It is thus possible that cells decode p53 signalling dynamics by integrating these different readouts - highlighting the need for multimodal measurements to deduce the logic of the cell decision-making process.

### 2.3 Response to growth factors: MAPK signaling

Another notable example of signaling system giving rise to rich dynamical behaviors is the MAPK (Mitogen-Activated Protein Kinase) family, which plays a crucial role in the regulation of various cellular processes like cell proliferation, differentiation and eventually death by transmitting signals from extracellular stimuli to intracellular targets (Boulton et al., 1990; Aoki et al., 2013; Rauch et al., 2016; Adlung et al., 2017; Goglia et al., 2020; Lavoie et al., 2020). MAPKs signaling cascade is frequently involved in oncogenesis and tumor progression and has consequently been thoroughly investigated over the past decades. The best characterized MAPK families are the ERK 1/2 (extracellular signal-regulated kinases 1 and 2), the JNK (c-Jun N-terminal kinases) and p38 (Cargnello and Roux, 2011; Braicu et al., 2019).

**FIGURE 4:**
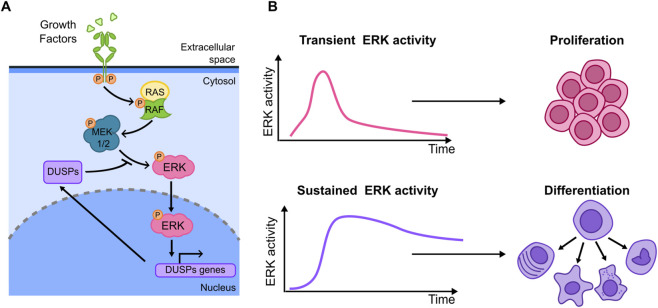
**(A)** Key elements of the ERK branch of the MAPKs signalling. **(B)** Different stimuli can lead to transient and sustained ERK activity (left). These in turn lead to distinct cell fates (right illustrations): transient activation has been linked to proliferation while sustained activation has been shown to promote cell differentiation in a number of cellular models.

The ERK signaling is mainly activated by tyrosine kinase receptors in response to growth factors (GFs) and insulin, leading to a series of phosphorylation events that transduce signals from the cell surface to the nucleus (Boulton et al., 1990; 1991; Shankaran et al., 2009) (Figure 4A). The ERK pathway is regulated by a network of feedback loops including negative regulators of ERK itself (Amit et al., 2007; Shin and Nguyen, 2017) such as DUSPs (Dual-Specificity Phosphatases). The first evidence of a relationship between ERK activation dynamics and cell fate comes from population-based experiments where it was shown how distinct ERK activation dynamics arising upon EGF (Epidermal Growth Factor) and NGF (Nerve Growth Factor) lead to different cell decisions. Using fixed-cell imaging techniques more than 30 years ago (Traverse et al., 1992) showed in epithelial PC12 cells that NGF-activated MAPK cascade led to a sustained (more than 1 h) ERK activation with plateau value reached in 5 min. On the contrary, EGF-induced ERK activation was even more rapid, with a peak value after 2 min, followed by a signal decrease to about 20% of the peak value in less than 20 min (Figure 4B). The authors then inferred that the different cell fate decisions triggered by these factors (with EGF leading to proliferation and NGF leading to neuronal differentiation) could be related with the distinct ERK activation dynamics induced by these stimuli (Traverse et al., 1992) (Figure 4B). This raised the possibility that ERK signaling dynamics might also play a role in the many other physiological and pathological conditions where this pathway is involved (Lavoie et al., 2020), a possibility that has been thoroughly tested at the single cell level by live cell imaging. (Ryu et al., 2015; Johnson and Toettcher, 2019; Guo et al., 2021; Ender et al., 2022; Bennett et al., 2024).

The richness of ERK activation dynamics at the single cell level was first probed in HEK293 cells using a FRET-based ERK activity reporter (Harvey et al., 2008). Discrete ERK activity pulses of fixed amplitude and 1 h period are observed at the single-cell level upon EGF stimulation and correlated with entrance in S phase (Albeck et al., 2013). Using a similar reporter in PC12 cells and modulating the frequency of ERK activation by delivery of EGF or NGF pulses at different frequencies with a microfluidics system (Ryu et al., 2015) showed that the neuronal differentiation fate (evidenced through morphological analysis of the cells) does not depend only on the GF used but also on the dynamics of administration, which in turn influences ERK accumulation dynamics. More recently, a study in PC12 cells, where ERK activation dynamics is controlled by electrical stimulation and monitored through live cell imaging, shows that also in this case different frequency, amplitude and duration of the modulation result in different ERK dynamics and hence different degrees of cell differentiation, assessed again through a morphological analysis (Guo et al., 2021).

Further evidence highlighted the role of ERK dynamics in cell differentiation during embryonic development, where differences in ERK activity are observed in different parts of the *Drosophila* embryo. Johnson and Toettcher focused on understanding how ERK signaling influences the first few hours of embryonic development and how different patterns of signaling correlate with distinct cell fates, employing optogenetic tools to generate different ERK activity profiles and observing their impact on development. By doing so, they could link the pattern of ERK signaling upon a given light stimulation to the expression of target genes specific to mesoderm or ectoderm identities, suggesting that spatial heterogeneity in ERK activation dynamics indeed contributes to cell differentiation. Hence, it is the precise temporal and spatial patterns of ERK activation that seems to determine embryonic cell fate partially through transcriptional mechanisms (Johnson and Toettcher, 2019).

In addition to its role in proliferation and embryonic differentiation, it has been proposed that non-periodic ERK pulses may control cell migration, quiescence and apoptosis during the development of the mammary gland in a 3D model of acinar morphogenesis (Ender et al., 2022). They showed that each of these phases is characterized by a specific ERK pulsing dynamics, including spatiotemporal coordinated states, which can be disrupted through optogenetic control. Importantly, pulsed ERK seems instrumental to avoid apoptotic cell fates: ERK pulses delivered at least every 4 h were able to promote survival in the acini cells (Ender et al., 2022).

ERK activity is often associated with Akt signaling (PI3K/Akt), a family of three Serine/Threonine protein kinases involved in cellular survival pathways (by inhibiting apoptotic pathways) and metabolism, in response to similar upstream activators like growth factors (Carnero et al., 2008). Dysregulation of both ERK and Akt pathways is one of the most frequent alterations observed in cancer cells, related to uncontrolled cell growth (Carnero et al., 2008; Setia et al., 2014; He et al., 2021), so their interplay has been investigated in different systems. In PC12 cells, using immunofluorescence, NGF stimulation revealed two subpopulations with distinct cell fates: one characterized by high ERK activation and low Akt activation, inducing cell differentiation, while the other exhibiting high Akt and moderate ERK activities associated with proliferation (Chen et al., 2012). Spatiotemporal control of ERK/Akt dynamics has also been suggested to play a role in tissue homeostasis in a recent study (Gagliardi et al., 2021). The authors observed ERK/Akt waves in 2D cell cultures starting from regions where apoptosis is induced. By directly correlating ERK signaling with cell fate, using a machine learning approach based on a high-content imaging dataset, they conclude that ERK signaling frequency determines collectively the balance between survival and apoptotic fates (Gagliardi et al., 2021).

A classification approach is also used in the works by (Stern et al., 2022; Bennett et al., 2024) to categorize dividing and non dividing MCF10 cells based on ERK/Akt signaling dynamics, starting from a training dataset. ERK dynamics *per se* are more predictive of cell division fate with respect to Akt, but accounting for their combined effect improves cell fate predictions. Similarly, the possible interdependence in determining cell fate decisions in ERK/Akt pathways has been explored in mESCs (mouse Embryonic Stem Cells) in (Arekatla et al., 2023). By means of imaging and optogenetic ERK and Akt manipulation, a counterbalance between the two has been suggested, with sustained ERK activation and decreased Akt activity marking the exit of stem cells from pluripotency, corroborated by changes in the expression of pluripotency markers.

In summary, the results from the last two decades evidence the relevant role of the ERK activation dynamics and of the ERK/Akt combined pathway in triggering specific cell decisions. Although the mechanisms used by cells to decode ERK/Akt have not been fully established, the possibility to manipulate ERK activation through optogenetics or using pulses of external stimuli (e.g., with EGF or NGF) represents a powerful tool to further investigate the role of signaling dynamics in cell decision making. We also believe that it is likely that similar approaches will contribute to our understanding of how other MAPK branches might influence cell fate decisions. Indeed, there is evidence showing that JNK activation dynamics might have a role in other cellular processes such as inflammasome formation and pyroptosis (Bradfield et al., 2023) and, related to p38 dynamics, in UV-C induced apoptosis (Miura et al., 2018). MAPK dynamics could also be important when considering simultaneous activation of other signaling pathways, as shown at single cell level when co-imaged with p53 (Hanson and Batchelor, 2022) or in the potential crosstalk between p38 and p53 involved in senescence (Freund et al., 2011). Machine learning approaches are evidencing how the dynamic crosstalk between MAPKs and other pathways seem fundamental also in tumor cells response to therapy (Netterfield et al., 2023).

### 2.4 Development and differentiation: The *HES* family and related pathways

As stated in the introduction, cell fate determination has predominantly been understood as the process by which cells differentiate into certain cell types, and is hence fundamental both in embryo development and in tissue homeostasis. The commitment of a cell towards a specific lineage during development is an evolutionarily conserved process that requires precise spatial and temporal control, as evidenced several years ago in cell transplantation experiments between Zebrafish embryos (Ho and Kimmel, 1993) or in *Drosophila* mutants (Heitzler and Simpson, 1991). In adults, stem cells converge to markedly different fates in organs such as the brain (Dray et al., 2021) or the intestine (Beumer and Clevers, 2021). For decades, stem cell fate decisions were shown to rely on a combination of cell-intrinsic (e.g., the expression of subsets of TFs) as well as cell-extrinsic (e.g., the morphogens gradient) factors (Valencia and Peter, 2024; Wang et al., 2024). However, in recent years growing evidence suggests that the activation dynamics of specific molecular players regulate cell fate choices in developing organisms and during differentiation. Here we will discuss some of the most thoroughly characterized TFs in this context: the Hes family members, master regulators of neurogenesis (Dhanesh et al., 2016), and YAP/TAZ, key factors in organ development (Moya and Halder, 2019).

**FIGURE 5:**
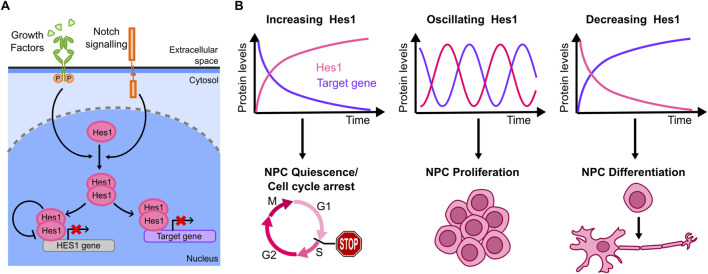
**(A)** Core of the Hes1 regulatory circuit, including its self-inhibiting negative feedback (other positive and negative feedback regulators have been reported). **(B)** Hes1 dynamics observed in the literature from live cell imaging include accumulation, oscillating dynamics and decreased levels (top), each with different effects on specific target genes. Correspondingly, distinct cell outcomes that have been associated with each of the Hes1 dynamics described above (bottom).

Hes1 (Hairy and Enhancer of Split-1) is a member of *HES* genes superfamily (Hes1-7), whose Hes1,3,5 members are mostly redundant and can compensate each other during neuronal differentiation (Kobayashi and Kageyama, 2014). Hes1 represents the prototype of this family and is the most characterized among the Hes factors. Hes1 is a TF that can be activated by either Notch-mediated juxtacrine signaling or *via* GFs paracrine signaling (Dhanesh et al., 2016) (Figure 5A). Once activated, it can homo- or heterodimerize with other TFs or cofactors to inhibit gene expression. Upon homodimerization, Hes1 directly binds and represses its target promoters; it can also repress transcription by sequestering specific transcriptional activators (Liu et al., 2015) (Figure 5A), including pro-differentiation genes (such as Ascl1, Neurog2, Neurod4 in nervous system), cell cycle regulators (cyclin E2/D1, p21) as well as Notch ligands (Dll1, Jag1) to maintain progenitors/stem cells pluripotency. Notably, dysregulation of the Notch-Hes1 axis sustains cancer progression by inhibiting differentiation and promoting cancer stem cell proliferation (Rani et al., 2016).

Hes1 exhibits an auto-inhibitory mechanism by binding to its own promoter and repressing its expression (Takebayashi et al., 1994) (Figure 5A). This leads to different dynamics - including oscillations - that have been proposed to be important to regulate downstream outputs (Figure 5B). The first evidence of Hes1 oscillatory dynamics and their association with the cell fate derives from bulk data. The auto-inhibitory circuit represents the hinge for generating Hes1 oscillatory behaviour, as seen by biochemical analysis correlating Hes1 protein and Hes1 mRNA levels over time in a cell model from mouse embryo (Hirata et al., 2002). Periodic expression of Hes1 was then associated with somite formation in mice embryos mesoderm, as probed by *in-situ* hybridization (Jouve et al., 2000). Importantly, only by studying these dynamics in individual cells using a bioluminescent reporter for live cell imaging, it was possible to demonstrate that Hes1 oscillations are synchronized in order to maintain specific levels in those cell lineages responsible for somite formation, (Masamizu et al., 2006). Similar approaches later revealed that Hes1 dynamics also play a role in neurogenesis.

Marinopoulou and collaborators (Marinopoulou et al., 2021) used NSCs (Neural Stem Cells) from transgenic mice carrying a similar luciferase reporter to study endogenous Hes1 dynamics: live-cell imaging showed that Hes1 oscillates both in proliferating and in quiescent cells, although with a longer oscillatory period in quiescent cells. Upon induction of a persistent ectopic Hes1 expression (which overrides endogenous Hes1 oscillations) quiescent cells fail to regain their proliferative capability (Figure 5B).

The mechanism connecting Hes1 oscillations to downstream effects in neuronal differentiation was partially clarified by (Imayoshi et al., 2013), again through live-cell imaging of a luciferase reporter: the downstream target Ascl1 (Achaete-Scute homolog 1, a TF controlling the expression of neuronal genes) display antiphasic oscillatory dynamics with respect to Hes1. When NPCs (Neuronal Progenitor Cells) undergo asymmetric division to generate a neuron and a NPC, Hes1 expression is lost in the neuron, causing Ascl1 protein accumulation in the daughter neuronal cell only. By controlling Ascl1 dynamics through optogenetics, the authors demonstrated that sustained Ascl1 expression leads to differentiation into neurons, while oscillations maintain the proliferative phenotype of NPCs (Figure 5B). Overall, this work provides compelling evidence that Hes1 oscillations prime the pluripotent cells to a specific lineage through the control of targets such as Ascl1.

Further mechanistic insights were provided by (Maeda et al., 2023) who focus on how *CDKN1A* (the gene encoding for p21) decodes Hes1 dynamics into cell fate decisions of NSCs using a bioluminescent marker and a FUCCI reporter, allowing to identify cell cycle phases in single cells. Upon Hes1 knock-out, cell proliferation was not significantly affected, due to compensation of other *HES* genes, but the knock-out of the whole *HES* family impaired cell proliferation. Conversely, upon ectopic sustained expression of Hes1, the number of cells arrested in G1 and G2 increased, indicating that an efficient cell proliferation is promoted by Hes1 oscillatory dynamics. RNA-seq data of wild-type *versus HES* family knock-out embryonic NSCs or *versus* Hes1-overexpressing cells showed that *CDKN1A* transcription was upregulated in both, while at the protein level p21 upregulation was observed in Hes1-overexpressing cells only. Optogenetic control of Hes1 levels further revealed that Hes1 oscillations promoted downregulation of *CDKN1A* expression after each pulse. This study thus highlighted that Hes1 dynamics, more than its levels, are fundamental to modulate p21 expression and hence the proliferation potential of NCSs. Finally, compelling evidence shows that Notch1 ligands Dll1 and Dll4 trigger distinct Notch1 activity dynamics leading to different Hes1 levels, which in turn determine cell fate decisions in embryonic myogenesis (Nandagopal et al., 2018). These results, together with the previous ones, highlight the importance of regulators of Hes1 dynamics in developmental cell fate decisions.

Dynamics have been also proposed to be relevant for other members of the *HES* family. For instance, Hes5 has been correlated with NSCs proliferation/differentiation in the developing mouse embryo (Bansod et al., 2017). NSCs differentiate less and proliferate more in Hes5-overexpressing brain cortex with respect to wild-type, where its expression is oscillatory and in phase with Hes1 (Imayoshi et al., 2013), and both Hes5-overexpressing and Hes5-knockout mice suffer from an aberrant brain development. Hes5 dynamics seem relevant, as spinal cord NPCs are more likely to differentiate into an interneuron when Hes5 displays damped oscillatory dynamics, while aperiodic Hes5 dynamics likely lead to differentiation into a motor neuron (Manning et al., 2019). Similarly to Hes1, Hes7 encodes for a transcriptional repressor following induction by Notch signaling. Hes7 was specifically discovered in PSM (Presomitic Mesoderm), where it regulates the segmentation clock during the development of the mouse embryo (Bessho et al., 2001a) and is also regulated by a self-inhibitory negative feedback loop (Bessho et al., 2003; Chen et al., 2005). Most studies have focused on the spatial patterning of Hes7 expression along the posterior-anterior axis during embryo development; if perturbed, it is known that it can lead to smaller PSM and somite irregularities (Bessho et al., 2001b; Chen et al., 2005; Niwa et al., 2007) but the temporal dynamics of Hes7 at single-cell resolution are only starting to be investigated (Matsuda et al., 2020).

Another important example of TFs whose dynamics have been connected to cell differentiation is provided by the HIPPO pathway effectors, YAP (Yes-Associated Protein) and TAZ (Transcriptional co-activator with PDZ-binding motif) (Huang et al., 2005; Reggiani et al., 2021). These factors have key roles in development, tissue homeostasis, stemness and proliferation (Kim and Jho, 2018), but are also found hyperactivated in several cancers (Zanconato et al., 2016). HIPPO pathway activation is followed by a kinase cascade leading to YAP/TAZ phosphorylation and nuclear export, resulting in their inactivation. The first hint about the importance of YAP/TAZ dynamics comes from a study of the segmentation clock during somite formation where the expression of a constitutive active form of YAP impaired the triggering of the gene expression waves needed for somite formation (Hubaud et al., 2017). Similarly persistent nuclear localization of YAP/TAZ impairs lung tissue differentiation as observed upon knockout of the negative regulators LATS1/2 in airway stem cells (Jeon et al., 2022).

Direct observations of YAP/TAZ dynamics at single-cell level, indeed revealed that YAP/TAZ nuclear concentration fluctuates and these dynamics correlate with transcriptional activation of downstream targets (Franklin et al., 2020; Koushki et al., 2023). Moreover, live-cell analysis from (Meyer et al., 2023) highlighted that the levels and timing of YAP control downstream target activation and cellular decision-making during development. Differentiating mESCs that lose their proliferative ability display an increasing fraction of oscillating and modulation of YAP dynamics through optogenetics is sufficient to differentially instruct cellular differentiation (which requires chronic low YAP levels) and proliferation (which instead requires oscillatory YAP dynamics) through regulation of Oct4 and Nanog expression.

Overall, the works discussed in this section provide strong evidence that also in proliferation, differentiation and tissue regeneration/homeostasis, both in adults and in embryos, the activity of transcription factors (*HES* family) and transcriptional regulators (YAP/TAZ) is exerted *via* specific dynamics of activation.

## 3 Discussion and conclusions

### 3.1 The growing evidence linking signaling dynamics and cell fate

The description of the selected signaling pathways above shows compelling evidence from different fields (from development to cancer therapy) of how a rich variety of signaling dynamics can provide predictive insights on the response of a cell to stimuli. Our selection of pathways might not be exhaustive and can have some bias.

First, in our effort to coherently organize the literature in major macro-areas we have left undiscussed other signaling pathways for which a role of signaling dynamics, characterized at single cell level, is being unveiled in cell fate decisions. An example is the SMAD pathway, for which an interesting pulsed dynamics of activation has been observed experimentally (Tidin et al., 2019) and has been convincingly related with proliferating/quiescence decisions in epithelial cells (Bohn et al., 2023). On the other hand, new studies are also pointing out to the role of signaling dynamics involving other transcription factors in contexts described above, as for NGN3 in embryo development (Miller et al., 2025). Since observation of complex signaling dynamics is becoming more widespread, we envision that insights on its role in different biological contexts will expand accordingly, and this might also improve our understanding of signaling-related diseases, like cancer (Yaffe, 2019).

Second, our description focused on circuits whose main feature is a negative feedback loop that might lead to oscillatory behaviour. However, for many of these systems, positive feedbacks have also been reported, that might lead to increased robustness (Tsai et al., 2008). Indeed, circuits dominated by positive feedbacks can also have a role in cell fate decisions, as evidenced by studies (not always imaging-based) in fungi (Huang et al., 2006; Zordan et al., 2006), mammalian cells (Wang et al., 2009; Tuvikene et al., 2016; Co et al., 2024), yeast (Zhang et al., 2017; Cerulus et al., 2018) and *Xenopus* (Xiong and Ferrell, 2003). Broadly speaking, these positive feedbacks lead to bistable behaviours (Ferrell, 2002). Hence, our interest in systems with a rich variety of dynamical behaviours - including oscillations - might have led to a bias in our selection of signaling systems. Nevertheless, the general principles outlined here would apply to this and other regulatory architectures like feed-forward loops, that here were discussed in the context of NF-κB (Metzig et al., 2020) and have recently been suggested to play a role in p53 as well (Dean et al., 2024).

Despite these potential biases, the examples discussed above highlight an important advantage of using live-imaging single-cell approaches: microscopy shows that even homogeneous populations of cells subjected to the same stimulus can display heterogeneous dynamics, and this allows studying directly the direct downstream effect of these different dynamics, with no confounding effects. Moreover, live-cell imaging has a unique ability of following individual cells over time, which is advantageous to identify causal relationships. Hence, it offers a valid complementary approach to single-cell sequencing techniques where temporally resolved single-cell trajectories towards specific fates cannot be directly assessed - although this can be partially overcome with the help of clever analysis methods such as RNA-velocity (La Manno et al., 2018; Riba et al., 2022).

**FIGURE 6:**
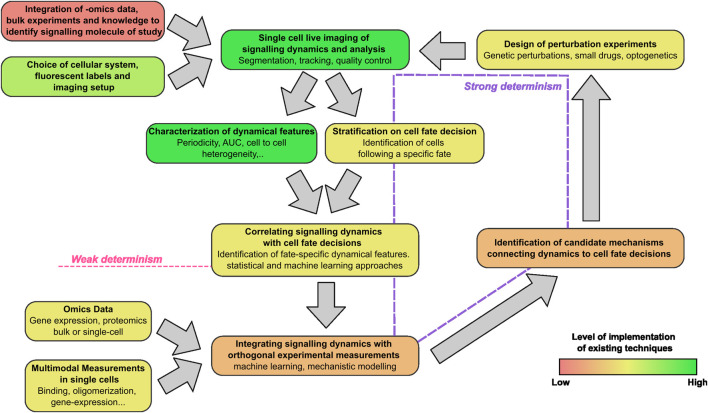
The live-cell imaging centered workflow to connect signaling dynamics and cell fate. The steps include the identification of the proper signaling dynamics to be characterized in each context, imaging and characterization of signaling dynamics, the connection with cell fate and the integration with orthogonal data (biophysical or omics). The color scale of the boxes from red to green indicates the current degree of development of each step in the workflow.

Taken together, we are witnessing the establishment of a research workflow based on live cell imaging that is providing novel clues on how cells make specific decisions in a wide variety of situations (Figure 6). This workflow includes steps that are fully attainable with existing technology and tools, such as imaging of the dynamics of interest and their characterization. Indeed, fluorescent labelling of proteins at endogenous level can now be performed routinely using gene editing approaches. Furthermore, libraries of fluorescently tagged cell lines where individual proteins are labeled at nearly endogenous levels are now available (Cho et al., 2022). At the same time, we have also identified specific steps of this workflow that represent challenges in the field, as we discuss in more detail below.

### 3.2 In what sense(s) can we say that signaling dynamics determine cell fate?

The examples presented suggest that there might be different ways to interpret the relationship between signaling dynamics and cell fate. We can essentially call them the *weak* and the *strong* deterministic sense. Data on signaling dynamics can be considered as a direct observation of the evolution of a subset of the variables that evolve in the process bringing the cell state from one attractor to a different one (Figure 1). From this partial observation of the trajectory linking the two states, we might be able to forecast the system evolution towards specific fates without having any insight on the mechanistic role of signaling dynamics in this process (Figure 6). It might even be possible that what we are observing does not play a direct role in the cell fate determination. As a theoretical example, even if the dynamics of a specific transcription factor *y* determines the expression of a protein *Y* that drives a specific fate, the direct observation of another target of *y*, called *Z,* expressed with similar kinetics to that of *Y*, would allow us to say that observing *Z* determines cell fate. Hence, for all the studies where a correlation between signaling dynamics and cell fate has been found we can say that signaling dynamics determine cell fate in a *weak sense*. Of note, recent studies show that distinct signaling dynamics can be faithfully mapped through mathematical models to specific changes in the parameters of the biochemical network governing them (Singh et al., 2024) that can be experimentally inferred from transcriptomics (Kizilirmak et al., 2023) or protein expression data (Yang et al., 2018). This opens the intriguing possibility that signaling dynamics and subsequent cell fate decisions could be itself predictable from this kind of data.

Dynamics can be said instead to determine cell fate in a *strong* sense if it is possible to prove that they are *causative* of the cell fate decisions. To this aim, we have shown that the usual approach is to design perturbation experiments aiming at selectively modulating the dynamics of the signaling molecules. These perturbations can be performed through genetic or pharmacological inhibition of regulatory circuits of the factor of interest ]e.g., the use of Nutlin-3a to inhibit the interaction between p53 and its negative regulator MDM2 (Purvis et al., 2012)], through the control of expression levels of the signaling molecules [e.g., by gene editing to generate inducible synthesis and degradation of the protein of study (Chassin et al., 2019)], microfluidics or optogenetic tools that allow spatiotemporal control of gene expression, protein localization or its aggregation (Toettcher et al., 2013; Shin et al., 2017; Strom et al., 2024). These approaches have been used to show that the cellular response to a stimulus depends not only on the expression levels of the signaling molecule, but also on the specific dynamics observed (e.g., oscillating vs non-oscillating) and the parameters describing these dynamics, such as the period of oscillations, their amplitude or the rate at which the protein of interest accumulates. Yet, as described above, in many contexts there is potential for a finer identification of the mechanisms linking signaling dynamics and cell fate decisions that could be achieved by the careful design of proper perturbation experiments able to prove causal mechanisms (Figure 6).

### 3.3 Is the dynamics of one signaling molecule enough?

Our review of the literature shows that correlations between signaling dynamics and cell fate decisions are never perfect. A simple explanation for this is due to the fact that signaling dynamics provides only a partial information of the global time evolution of the system towards an attractor (Figure 1A). From a purely theoretical dynamical point of view observing only one variable has only been shown to be sufficient to reconstruct completely the dynamics of a very specific class of deterministic systems with complex (chaotic) behaviours (Takens, 1981). However, it is easy to find examples of dynamical systems where the observation of a single variable is not enough to reconstruct the whole dynamics. For example, consider the movement of a particle in a two-dimensional potential V (x, y) symmetric along x and y-axis [V (±x,±y) = V (x,y)] with four local minima in the points (±a,±b) and in presence of friction, so trajectories eventually settle to any of the 4 attractors (fates). It is then easy to see that trajectories can settle to different attractors (with y coordinate equal to either b or -b) even if the evolution of *x(t)* is identical, so a single variable [x(t)] does not determine the y variable (nor the system’s “fate”).

Hence, an important limitation of live-cell imaging is its targeted nature, and the difficulty in scaling those experiments to analyze multiple signalling molecules and pathways. However, the body of work described in this review provides encouraging evidence suggesting that a limited number of signalling systems can be predictive of cell fate decisions. Additional experimental evidence provides further support for this notion. A recent theoretical analysis of single-cell RNA-seq experimental data from development suggest that cell fate transitions are essentially governed by low-dimensional dynamical systems, where the variables are specific combinations of certain protein abundances (Sáez et al., 2022). Along these lines, a clever study that infers TF activity from single-cell RNA-seq (featuring a novel method that combines RNA-velocity approaches and genomic information) suggests that a relatively low number (10–20) of transcription factors are significantly activated in beta-cell differentiation (Jiménez et al., 2023). This is aligned with the works where different signaling dynamics are observed simultaneously. We already discussed how both the “competition” in the accumulation dynamics of p53 and inhibitors of apoptosis are determinants of cell death (Paek et al., 2016). As an additional example simultaneous recording of p65 and p38 dynamics has recently allowed to identify the amount of information that can be carried by each signaling pathway upon their co-activation by pro-inflammatory cytokines, and their contribution to heterogeneity in downstream transcription (Luecke et al., 2024). Overall, we expect that imaging of more than one signaling pathway at a time might drastically improve our ability to establish connections with cell fate decisions.

### 3.4 The challenge of integrating signaling dynamics data with omics and biophysical data

Considering that potentially a limited number of signaling systems might be determinant of cell fate decisions, a more profound integration between single-cell and bulk omics data could render the choice of the pathways and proteins whose dynamics should be followed less biased and more informative. For example, transcriptomics at multiple time points following a stimulus could be used to identify those proteins displaying interesting dynamics (e.g., oscillations), that could be then studied more in detail by live-cell imaging; similarly, omics approaches are able to establish key pathways involved in cell to cell communication and then help identify those whose dynamics are worth exploring (Armingol et al., 2024) (Figure 6). We also speculate that it might be possible to infer single cell signaling dynamics data from high-resolution multi-modal omics datasets; however, to achieve this goal it would be necessary to perform carefully designed experiments where the inferred dynamics could be compared with experimentally measured ones (e.g., by performing live cell imaging data and multi-modal data analysis in the same cells), and would also require advanced computational techniques - possibly model informed-able to reconstruct signaling dynamics from static high-throughput omics data.

Further, complementary experimental advantages could derive from measuring multiple biophysical properties of the signaling molecules studied: signaling typically involves not only dynamic changes in the amount or localization of the factor of interest, but also in the accumulation of post-translational modifications, homo- or hetero-oligomerization and–in case of transcription factors–the modulation of interactions with DNA. Different live-cell fluorescence microscopy techniques can be used for measuring, with single-cell sensitivity, these parameters. For example, Number&Brightness approaches have been used to probe that p53 rapidly oligomerizes in single cells in response to DNA damage, and that p53 oligomerization is sufficient to induce the activation of p53 (Gaglia et al., 2013). Similarly, we used single molecule tracking methods to demonstrate that the fraction of p53 molecules engaged in chromatin binding follows itself oscillatory dynamics upon DNA damage, and that more stable p53-DNA interactions are associated with stronger transcriptional outputs (Loffreda et al., 2017). Finally, the recent development of intrabodies, antibody fragments expressed by the cell of study, might soon enable monitoring how dynamically signaling molecules are post-translationally modified in living cells (Galindo et al., 2025) and this has been recently proposed to be a fundamental step to provide dynamic-specific transcriptional responses by a combination of experiments and mathematical models in yeast (Sweeney and McClean, 2023). These advanced techniques are typically applied in isolation, one at the time, but we foresee that their future integration will advance our understanding of the molecular details linking signaling dynamics to phenotypic outcomes. Integration of these orthogonal measurements with single-cell live cell imaging data remains to be fully developed and will require additional experimental and theoretical efforts (Figure 6).

### 3.5 Beyond (downstream) signaling dynamics: insights and potential limitations

From our review of the literature it emerges that identifying the precise biophysical and molecular mechanisms that underline the causal relationship between dynamics and cell fate remains an open challenge. In many of the works cited above cell fate determination arises through the activation of dynamics-specific gene expression programs. These have been experimentally demonstrated in a number of studies (Hao et al., 2013; Sen et al., 2020; Sweeney and McClean, 2023; Lu et al., 2024), presumably involving a trade-off between noise and control of gene expression (Hansen and O’Shea, 2013). A complete picture on the fine molecular details and on the integration of signalling dynamics within the different timescales involved in gene-expression regulation [TF binding to the promoter (Mazzocca et al., 2021), activation of the transcriptional machinery (Meeussen and Lenstra, 2024), and chromatin modifications (Saxton et al., 2023)], though, is still lacking, Of note, this should include the existing evidence that points to a role of the dynamics of transcription and translation (including degradation timescales) in providing specific gene expression programs (Porter et al., 2016; Zambrano et al., 2016; Hanson et al., 2019).

On the other hand, experimental evidence suggests that there might be fundamental limits in our ability to establish one-to-one correspondence between signaling dynamics and transcriptionally mediated cellular outcomes, since some of the molecular processes involved in decoding those dynamics can be intrinsically stochastic (Losick and Desplan, 2008). The transcription of a gene is a clear example (Raj and van Oudenaarden, 2008; Symmons and Raj, 2016). A given gene is only present in a few copies in each nucleus, and its activation depends on TF molecules finding those targets in a transcriptionally permissive state, leading to transcriptional bursting. Consequently, two identical cells could display different transcriptional responses to similar dynamics of the same TF. Methods to probe transcriptional bursting in living cells, like those based on the imaging of MS2 reporters (Pichon et al., 2018), exist, but only a few studies attempted to correlate dynamics of the activating TF to the transcriptional kinetics of its targets at the single cell level, showing varying degrees of stochasticity. Transcription of the p53 target *CDKN1A*, for example, seems to be moderately affected by intrinsic stochasticity: every pulse of p53 accumulation in response to DNA damage leads to *CDKN1A* transcription in nearly every cell (Hafner et al., 2020). Differently, an MS2 reporter for NF-κB mediated transcription displayed a poor correlation between p65 dynamics and downstream transcriptional kinetics (Zambrano et al., 2020). We expect that further studies will clarify the influence of intrinsic stochasticity on the activation of other transcriptional targets controlled by these and other signaling molecules. Integrating data on multiple transcriptional targets - combining live-cell measurement of signaling dynamics and transcriptional readouts at endpoints by multiplexed smFISH (Lee et al., 2014) or sequencing - could help find quantitative relations between signaling dynamics and transcription. Furthermore, they could contribute to identify if mechanisms at the gene network level exist to buffer stochasticity at the single gene level. A similar approach has been recently used to estimate the amount of information carried by Ca^2+^ signaling that is transmitted to downstream transcription (Foreman and Wollman, 2020), highlighting that signaling dynamics, cell size and cell cycle stage are major determinants of the variability in the cell transcriptional response.

### 3.6 Concluding remarks

The identification of the molecular mechanisms linking signaling dynamics to cellular outcomes will likely require combining information from multiple pathways and to account for the inherent stochasticity underlying transcriptional regulation and gene expression. These two aspects might need to be tackled simultaneously, as we cannot exclude that concomitant activation of different signaling mechanisms can confer some sort of buffering able to provide convergent response even in presence of stochastic gene expression. Indeed, to limit the biological variability caused by the factors aforementioned, complex buffering mechanisms have been reported in signaling systems like those governing development, where Notch oscillations in isolated embryo cells are asynchronous but become synchronized in the developing tissue, leading to tighter gene expression control (Tsiairis and Aulehla, 2016). Overall, we think that the theoretical possibilities sketched above might well be confirmed (or dismissed) in the next few years, where it is foreseeable that more studies will be based on multiplexed investigation of signaling dynamics, presumably also *in vivo*. Here, the concomitant development of theoretical models and data analysis pipelines (potentially AI-assisted) to recognize, isolate and select dynamic phenotypes will be fundamental to dissect how the cell decodes different temporal and spatial evolutions of signaling molecules into specific cell fates.
